# The determinants of the quality of clinical management among diabetic and hypertensive patients in a context of fragility: A cross-sectional survey from Lebanon

**DOI:** 10.3389/fpubh.2022.844864

**Published:** 2022-07-25

**Authors:** Shadi Saleh, Dina Muhieddine, Randa Hamadeh, Hani Dimassi, Karin Diaconu, Stella Arakelyan, Alastair Ager, Mohamad Alameddine

**Affiliations:** ^1^Global Health Institute, American University of Beirut, Beirut, Lebanon; ^2^Department of Health Management and Policy, Faculty of Health Sciences, American University of Beirut, Beirut, Lebanon; ^3^Ministry of Public Health, Beirut, Lebanon; ^4^Department of Pharmaceutical Sciences, School of Pharmacy, Lebanese American University, Byblos, Lebanon; ^5^Institute for Global Health and Development, Queen Margaret University, Edinburgh, United Kingdom; ^6^College of Health Sciences, University of Sharjah, Sharjah, United Arab Emirates

**Keywords:** non-communicable diseases, diabetes, hypertension, fragility, refugees, equity, Lebanon

## Abstract

**Introduction:**

The management of NCDs is a growing challenge in low- and middle-income settings with the increasing prevalence and the associated demands that such conditions make on health systems. Fragile settings both exacerbate the risk of NCDs and undermine systems capacity. Lebanon is a setting where strategies to address rising NCDs burden have faced particularly acute contextual challenges.

**Methods:**

We conducted a cross-sectional survey with patients accessing non-communicable disease across 11 primary care centers within the Greater Beirut and Beqaa areas. Response were received from 1,700 patients. We generated a Clinical Management Index Score as a measure of quality of care, and scores related to a range of socio-demographic characteristics and other context specific variables.

**Results:**

Significantly higher clinical management index scores (better quality of care) were associated with patients living in the semi-urban/rural context of Beqaa (compared to Greater Beirut), having health insurance coverage, aged above 60, having high levels of educational attainment, and making partial or full payment for their treatment. Relatively lower index scores (poorer quality of care) were associated with Syrian nationality (compared to Lebanese) and with patients suffering from diabetes or hypertension (compared to comorbid patients).

**Conclusion:**

The study identified a wide margin for improving quality of NCDs care in fragile contexts with particular gaps identified in referral to ophthalmology, accessing all prescribed medication and receiving counseling for smoking cessation. Additionally, findings indicate a number of predictors of comparatively poor quality of care that warrant attention, notably with regard to Syrian nationality/legal status, lack of health coverage, seeking free health provision and lower educational attachment. Although these are all relevant risk factors, the findings call on donor agencies, NGOs and provider institutions to design targeted programs and activities that especially ensure equitable delivery of services to diabetic and hypertensive patients with compounded vulnerability as a result of a number of these factors.

## Introduction

Non-communicable diseases (NCDs) including cardiovascular diseases, diabetes, chronic respiratory diseases and cancer are the leading causes of mortality worldwide (1). In Lebanon, NCDs contribute to 91% of all deaths and cardiovascular diseases alone account for almost half of NCDs mortality (2). The majority of premature deaths due to NCDs occur in low and middle-income countries (LMICs) (3). LMICs disproportionately suffer from NCDs, especially that those countries are hosting the greatest proportion of conflicts in the world (4). Patients in conflict and post-conflict settings are often more vulnerable to NCDs due to the increase in negative coping mechanisms, which often constitute NCDs risk factors, such as smoking and alcohol consumption. The growing burden of NCDs in LMICs aggravates existing health threats and worsens poverty and presents substantive challenges to the equitable delivery of affordable care (4, 5).

Fragility is commonly defined as “the combination of exposure to risk and insufficient coping capacity of the state, system and/or communities to manage, absorb or mitigate those risks” (2). Hence, fragility exposes populations to a range of threats including poverty, insecurity, as well as social and economic inequality (6). There is a huge gap in access to health services in countries experiencing fragility (7). Around 1.3 billion people globally have no access to effective and affordable health care, and among the latter, about 170 million spend more than 40% of their income on health expenditure (8).

The management of chronic diseases represents a major challenge for healthcare systems globally, given that they often require a long period of supervision and observation or care (9). Inadequate management of chronic diseases represents an important factor in the development of adverse outcomes, including hospitalizations (10). Factors that contribute to the poor management of chronic diseases include low educational and income levels among patients, strained patient-provider relationships, limited or non-existent health insurance or health coverage. Patients suffering from chronic diseases face increased healthcare utilization costs, decreased self-reported health status, and reduced functional capacity (11).

There is a dearth of data on care quality in LMICs, particularly in areas such as system competence, confidence in the system, and user experience and wellbeing, including patient-reported health outcomes (12). Health systems often produce inadequate insight on what matters most to people, such as competent care, user experience, health outcomes, and confidence in the system (12). Furthermore, few studies have investigated the associations between self-reported patient satisfaction scores and health outcomes (e.g., physical health, hospital utilization, and expenditures) while accounting for baseline patient-level characteristics (e.g., gender, education level, SES) (13).

### The local context

Lebanon is an upper middle-income country in the Eastern Mediterranean Region with an estimated population of over 6 million (14) of which more than one million are Syrian refugees (15). The Lebanese healthcare system is highly privatized, with healthcare delivery primarily provided by the private sector (16). Six social insurance funds cover health needs under the tutelage of different government bodies (17), they include: The National Social Security Fund (NSSF), The Civil Servants Cooperative (CSC), and the four military schemes (18). Despite of these funds, almost half of the population remains without any formal health coverage (19). The Lebanese civil war had an enormous negative impact on the public health care system, which was further exacerbated following the massive influx of Syrian refugees following the Syrian crisis (20).

The burden of NCDs remains the largest in Lebanon (5). Considering the local unrest, economic crisis, COVID-19 pandemic, Beirut Port explosion and other substantial challenges the country is facing, Lebanon has become more vulnerable to fragility, especially in rural and semi-urban areas where the health system and support services are relatively weak (21, 22). The aforementioned challenges made providing comprehensive NCDs care for the entire patient population a significant challenge especially that patients are of various nationalities and fall under diverse health coverage schemes. For instance, 58% of the Syrian refugees not receiving financial support from Non-Governmental Organizations (NGOs) access Primary Health Care Centers (PHCCS) for NCDs care compared to 17% of the host community (23).

Lebanon has been left stumbling following a financial crisis that hit the country since 2019 and worsened as the Lebanese pound lost 81% of its value (52). In the middle of this economic and political instability, the health care system continued to struggle with a shortage of supplies and medication and an exponential increase in the number of patients (53), the government was not able to set aside a stimulus package for hospitals to aid in supplies and resources as the pandemic surged and as a result some hospitals sustained depending on international and local non-governmental aid such as the WHO and NGOs to import essential supplies and equipment (53). In addition, as a consequence of the tremendous devaluation in the country's currency, the pay for a physician in Lebanon has dwindled to an estimated total loss in physicians' income by more than 80% (54) which lead to an enormous exodus of health care professionals, with almost 40% of skilled medical doctors and almost 30% of registered nurses leaving the country (55). All that combined negatively impacted the quality of care, and if no proper action is to be taken, the Lebanese healthcare system is expected to collapse.

This study assessed the differences in the quality of clinical management among diabetic and hypertensive patients accessing PHCCs in two different fragile settings in Lebanon. Differences in clinical management were related to sociodemographic factors such as age, gender, nationality, setting, and health status.

## Methods

The current study received ethical approval from the Institutional Review Board (IRB) for Social and Behavioral studies at the American University of Beirut—Protocol number SBS-2018-0514. It also received ethical approval from the IRB committee at the Queen Margaret University (Protocol number REP 0201).

### Study design and setting

This was a cross-sectional study using a quantitative survey design, conducted in two contrasting regions of Lebanon between January and July 2020. The two regions were the urbanized area of Greater Beirut (fragility setting 1) and the Beqaa Valley (fragility setting 2), see Box 1. Data collection occurred in two phases. The first phase extended from February to March 2020 during which the research team collected data from 708 patients. The mounting insecurity and restrictions imposed due to COVID-19 forced a temporarily suspension of data collection with the remaining 992 patients recruited between June and July of 2020.

Box 1 **Setting information.**

**Table:** 

**Fragility context one: the greater beirut area**	**Fragility context two: the beqaa valley area**
The main urban commercial center of the country, accommodating a population of 2,434,609, including 206,628 Syrian and 17,486 Palestinian and other nationalities (24). Has the highest concentration of health services available in the country, including access to specialized secondary and tertiary care services. Has high levels of socio-economic inequality, which has worsened since the damage experienced by the Beirut Port explosion in August 2020, which left 300,000 people homeless (25).	Predominantly a rural environment, where the main economic activity is focused on agricultural industry. Accommodates the highest number of Syrian refugees settled in Lebanon (339,473, 38.6% % of whole refugee population) (24). In contrast to Beirut, the health system in the Beqaa has been historically under-developed and under-resourced: only 22 public primary healthcare centers (26) and 21 hospitals are available in the region (27), with limited access to secondary and tertiary care and referrals forwarded to Beirut.

A total of eight data collectors were recruited. Data collectors attended a 2-day training which included an overview of the study and its objectives, the recruitment process, and research ethics and proper surveying practices. Data collection was performed using KoBo, a toolkit for collecting and managing data in challenging environments (28).

### Participants

Targeted health facilities were PHCCs, highly accessed by Lebanese and Syrian populations, that offered diabetes and hypertension services. Overall, 14 PHCCs were approached, of which 11 agreed to participate in the study. At these facilities, targeted participants were Syrian or Lebanese individuals who were: (1) older than 40 years, and (2) diagnosed with diabetes or hypertension (based on personal self-reporting of a confirmed physician diagnosis). Patients not meeting the above-mentioned criteria or not consenting to participate were excluded.

### Data collection

Eligible patients receiving NCDs services were approached before attending their appointment. The survey required ~40 min to be completed. Items on clinical management were self-reported; sections related to laboratory testing were completed with reference to documentation regarding the laboratory results that patients are required to bring with them to every appointment.

The study questionnaire was reviewed by an expert panel including health services/system researchers and clinicians from Lebanon and the United Kingdom. The local context was taken into consideration and necessary amendments were introduced through a group consensus process. The final draft of the questionnaire was translated to the Arabic language by an expert translator and back-translated to English by another translator. The original and back-translated English versions were compared, and some minor edits were introduced to the translated version to account for any differences and ensure the accuracy of translation. The final Arabic version of the questionnaire was pilot tested on 40 patients. Their feedback on the clarity, readability, comprehensiveness, and completion time required was solicited. Patients' feedback included the need for explaining some medical terms used in the questionnaire and the merging of some redundant questions for diabetic and hypertensive patients to make the survey shorter. The Arabic version of the questionnaire was modified in light of the feedback received during pilot testing.

### Sample size

Sample size was determined based on the primary research aim of determining differences in the quality clinical management score between groups. The sample size was based on a planned Analysis of Variance (ANOVA) with 5 groups. The minimum required sample was calculated as 1,550, based on a desired power set at 90%, a type I error set at 5%, and an effect size Cohen's F of 0.1 (29). The determined sample size was deemed appropriate for analysis of other dependent variables: hospitalization, visits to physicians, and lab tests. A total of 1,700 participants were recruited.

### Data sources

The survey questionnaire comprised 76 questions distributed across 7 sections as per Table 1.

**Table 1:** Description of questionnaire sections and sources.

**Section**	**# of questions**	**Description**	**Source**
1	12	Socio-demographics characteristics	World Health Organization (WHO) individual questionnaire (30)
2	11	disease risk factors	WHO individual questionnaire (30)
3	6	history of disease	WHO individual questionnaire (30)
4	26	itemized accounts of services received at (PHCC) or *via* referral	World Bank package (30)
6	1	General access to services	Drafted by the research team
7	1	Affordability of NCDs services and care coverage	Drafted by the research team

### Clinical management index scoring

We generated the quality of care (QoC) 10-item index to assess the quality of health services offered to diabetic and hypertensive patients at primary care clinics in Lebanon. The selection of 10 items was informed by the World Health Organization (WHO) Package of Essential NCDs interventions for primary care in low-resource settings (31), literature on the recommended management of diabetes (32), and the World Bank Basic Package of Health Services (33) adopted by the Lebanese Ministry of Public Health (MoPH) at the primary care level. Further consultations were held with local experts to check the appropriateness of the QoC measure to the Lebanese context. The index was constructed by summing the points from binary items (yes = 1, no = 0) asking about a minimum of 2 annual consultations with a General Practitioner (GP), referrals and attendance to appointments with an ophthalmologist, administration of laboratory tests (lipid profile test, Hba1c test, spot urine micro-albumin), lifestyle counseling, medication prescription and collection. The QoC index ranges from 0 to 10, where higher values indicate better alliance with the recommended national guidelines on diabetes and hypertension management and control.

### Statistical methods and main variables

Data was analyzed using Statistical Package for the Social Sciences (SPSS) v27 (34). The main dependent variable was clinical management which was assessed as a score ranging from 1 to 10 using a set of clinical items ranging from visits to a general practitioner to prescribed and received medication (see following sections). The items are in line with WHO Package of Essential Non-communicable disease (PEN) recommendations and have been reviewed by local MoH experts. The score was deemed to have a normal distribution after checking its histogram, Quantile-Quantile (QQ) plot, and skewness and kurtosis scores. Differences in clinical score were tested using either the independent *t*-test (for two groups) or the ANOVA F test. Variables that showed statistical significance at the bivariate level in the previous step were included in a multivariable linear regression with clinical management as the outcome.

## Results

### Characteristics of the study population and performance

The majority of participants were females (67.3%), Syrian (58.1%), married (81.6%). 39.8% were aged over 60. The majority of participants were drawn from setting 2 (84.1%). Just over half of the participants reported having no health coverage (51.8%). The majority of participants were hypertensive (53.4%), 26.9% were diabetic and 19.6% were comorbid. 43.6% of the respondents had no formal schooling and another 37% had only completed primary education. Only 14.5% of respondents reported being employed and the rest were either unemployed (79.3%) or unable to work (6.2%) (Table 2).

**Table 2:** Characteristics of the study population.

**Characteristics**	***N*** **(%)**
**Age groups**	
≤ 49	432 (25.4%)
50–59	591 (34.8%)
60 +	676 (39.8%)
**Gender**	
Female	1,144 (67.3%)
Male	556 (32.7%)
**Nationality**	
Lebanese	712 (41.9%)
Syrian	988 (58.1%)
**Marital status**	
Single	56 (3.3%)
Married	1,387 (81.6%)
Divorced/widowed	256 (15.1%)
Fragility setting*	
Setting 1	270 (15.9%)
Setting 2	1,430 (84.1%)
**Education**	
No formal schooling	741 (43.6%)
Primary	633 (37.3%)
Secondary	193 (11.4%)
High school and above	132 (7.7%)
**Employment**	
Working	247 (14.5%)
Not working	1,347 (79.3%)
Unable to work	105 (6.2%)
**Health coverage**	
No	874 (51.8%)
Yes	812 (48.2%)
**Health condition**	
Diabetic	458 (26.9%)
Hypertensive	908 (53.4%)
Comorbid	334 (19.6%)

^*^The urbanized area of Greater Beirut (fragility setting 1) and the Beqaa Valley (fragility setting 2).

### Performance of the study population on the clinical management index score

Out of all patients, 61.4% reported attending two GP visits over the last 12 months, with diabetic patients reporting the highest proportion (67.2%) and hypertensive patients the lowest (57.7%). While comorbid patients were the most referred to ophthalmologists (26%), hypertensive patients were least referred (9.8%). When referred, patients were strongly adherent, with 93% of patients reporting attending their referral to ophthalmologist consultations. Comorbid patients were the most tested for lipid profile (83.4%) and diabetic patients the least tested (65.5%). Unsurprisingly, diabetic patients were most tested for Hba1c (94.8%) and hypertensive patients were least tested (40.1%). Urine tests were performed by more diabetic (66.8%) and comorbid patients (66.5%) compared to hypertensive patients (58.3%). Across conditions, hypertensive patients were the least likely to have received nutrition advice (78.9%), but the most likely to report receiving most to all prescribed medications (18.5%). Comorbid patients were the most likely to receive smoking cessation advice and prescribed medications (Table 3).

**Table 3:** Description of items for clinical management score for hypertensive and diabetic patients.

**Items**	**All patients**	**Diabetic**	**Hypertensive**	**Comorbid**	***P*** **-value**
	***N*** **(%)**	***N*** **(%)**	***N*** **(%)**	***N*** **(%)**	
Offered/attended 2 GPs visits	1,042 (61.4%)	307 (67.2%)	523 (57.7%)	212 (63.7%)	0.002
Referred to an ophthalmologist	288 (16.9%)	112 (24.5%)	89 (9.8%)	87 (26%)	<0.001
Attended ophthalmologist	272 (94.8%)	105 (93.8%)	85 (96.6%)	82 (94.3%)	0.674
**Tests requested**					
Lipid profile	1,202 (70.9%)	300 (65.5%)	625 (69.0%)	277 (83.4%)	<0.001
Urine test	1,057 (62.2%)	306 (66.8%)	529 (58.3%)	222 (66.5%)	0.002
HbA1c	1,099 (64.8%)	434 (94.8%)	363 (40.1%)	302 (91.0%)	<0.001
Smoking advice	665 (44.2%)	171 (40.7%)	348 (44.2%)	146 (48.8%)	0.097
Nutrition advice	1,330 (85.6%)	421 (92.9%)	610 (78.9%)	299 (91.2%)	<0.001
Prescribed medications	1,221 (91.9%)	235 (86.1%)	659 (91.3%)	327 (98.2%)	<0.001
Received most to all medications	254 (15.7%)	42 (9.3%)	156 (18.5%)	56 (17.4%)	<0.001

*GP, General practitioner*.

### Clinical management index score by patient characteristics

Overall, the average clinical management index score was 5.7 (out of 10) across all patient groups. Comorbid patients had the highest score compared to diabetic and hypertensive patients (6.62 ± 1.7 vs. 6.13 ± 1.78 and 5.15 ± 1.94, respectively) (*p* < 0.001). Clinical management index scores were significantly higher among the age group older than 60 (5.96 ± 1.91) (*p* < 0.001) compared to lower age groups (5.69 ± 1.88 for age groups 50–59 and 5.32 ± 2.04 for age group under 49) (*p* < 0.001). Scores for females were significantly lower than for males (5.55 ± 1.96 vs. 6.02 ± 1.89) (*p* < 0.001). Scores for Lebanese patients were significantly higher than for Syrians (6.03 ± 1.95 vs. 5.46 ± 1.92) (*p* < 0.001). Scores also differed by setting, with higher scores in Beqaa/setting 2 (5.72 ± 1.93) compared to Beirut/setting 1 (5.6 ± 2.06). Bivariate analysis revealed no significant differences by health coverage status. Patients with no formal schooling scored significantly lower (5.47 ± 1.96) compared to patients with higher education levels (*p* < 0.001). Generally, clinical management scores were significantly higher for patients who contributed partially or fully to the payment of consultation items (i.e., consultation, medications, diagnostic tests, etc.) than those who did not contribute (*p* < 0.001). Patients who paid fully or partially for their medications scored significantly higher compared to those who received their medications for free (6.13 ± 1.94 vs. 5.46 ± 1.92) (*p* < 0.001), with similar results observed for patients who paid partially or fully for consultations (5.85 ± 1.89 vs. 5.57 ± 2.0) and diagnostic tests (5.93 ± 1.81 vs. 5.55 ± 2.05) (*p* < 0.001) (Table 4).

**Table 4:** Clinical management index score by patients' characteristics and financial arrangements.

	**Clinical management index score (0–10)**	
	**Mean (SD)**	***P*** **-value**
**OVERALL**	5.70 (1.95)	
**PATIENTS' CHARACTERISTICS**		
Health condition
Diabetic	6.13 (1.78)	
Hypertensive	5.15 (1.94)	
Comorbid	6.62 (1.70)	<0.001
**Age groups**		
≤ 49	5.32 (2.04)	
50–59	5.69 (1.88)	
60 +	5.96 (1.91)	< .001
**Gender**		
Female	5.55 (1.96)	
Male	6.02 (1.89)	<0.001
**Nationality**		
Lebanese	6.03 (1.95)	
Syrian	5.46 (1.92)	<0.001
**Fragility setting**		
Setting 1	5.60 (2.06)	
Setting 2	5.72 (1.93)	0.356
**Health coverage**		
No	5.66 (1.94)	
Yes	5.75 (1.96)	0.337
**Education level**		
No formal schooling	5.47 (1.96)	
Primary	5.83 (1.95)	
Secondary	6.11 (1.83)	
High school and above	5.80 (1.90)	<0.001
**Proportion of medicine expenses paid by patient**		
Free	5.46 (1.92)	
Partial/full	6.13 (1.94)	<0.001
**Proportion of consultations paid by patient**		
Free	5.57 (2.02)	
Partial/full	5.85 (1.89)	0.004
**Proportion of diagnostic tests paid by patient**		
Free	5.55 (2.05)	
Partial/full	5.93 (1.81)	<0.001
**Patients payment contribution**		
None	5.21 (2.02)	
Partial to full	5.96 (1.88)	<0.001

At the multivariate level, once all variables were accounted together in a regression model, fragility setting 2, having health coverage, age above 60, primary and secondary educational levels, and partial or full payment contribution were all are associated with a higher clinical management index score. In contrast, being of Syrian nationality, and suffering from diabetes or hypertension were associated with a lower index score. On average those living in fragility setting 2 had a higher score index by 0.94 points (*p* < 0.001) compared to their counterparts in fragility setting 1. Patients 60 years old and older received an average 0.442 higher points on the quality of clinical management scale compared to patients below the age of 50 (*p* < 0.001). Patients with primary education (*b* = 0.265, *p* = 0.011) and secondary education (*b* = 0.513 *p* = 0.001) were significantly more likely to receive better clinical management score compared to those without formal schooling. Furthermore, patients who partially or fully pay for services reported significantly better clinical management scores compared to those who received services for free (*b* = 0.688, *p* < 0.001). Syrian patients received significantly lower clinical management scores (*b* = −0.55, *p* < 0.001) compared to their Lebanese counterparts. Results also show that, compared to comorbid patients, diabetic patients (*b* = −0.538, *p* < 0.001) and hypertensive patients (*b* = 1–0.445, *p* < 0.001) reported a significantly lower clinical management index score (Table 5).

**Table 5:** Multivariable linear regression model for clinical management score index by patient characteristics.

	***B***	**Std. Error**	***P*** **-value**
(Constant)	4.48	0.353	<0.001
**Fragility setting**			
Setting 1 (reference)	–	–	
Setting 2	0.943	0.151	<0.001
**Health coverage**			
No (reference)	–	–	
Yes	0.438	0.096	<0.001
**Gender**			
Female	–	–	
Male	0.118	0.101	0.239
**Age groups**			
≤ 49 (reference)	–	–	
50–59	0.252	0.117	0.032
60 +	0.442	0.120	<0.001
**Nationality**			
Lebanese (reference)	–	–	
Syrian	−0.550	0.105	<0.001
**Education level**			
No formal schooling (reference)	–	–	
Primary	0.265	0.105	0.011
Secondary	0.513	0.156	0.001
High school and up	0.348	0.195	0.074
**Health condition**			
Comorbid (reference)	–	–	
Diabetic	−0.538	0.136	<0.001
Hypertensive	−1.445	0.122	<0.001
**Patients payment contribution**			
None			
Partial to full	0.688	0.100	<0.001

*Adjusted R-square = 17.1%*.

## Discussion

This is the first of its kind study in Lebanon examining the quality of the clinical management of diabetic and hypertensive patients at PHCCs in the fragile context of Lebanon. The study found that a significantly higher clinical management index score was associated with patients: living in the semi-urban/rural areas (setting 2/Beqaa), with health coverage, aged above 60, having primary and secondary educational levels, and making partial or full payment for their treatment. In contrast, significantly poorer quality of clinical management index scores were associated with Syrian nationality (compared to Lebanese) and with patients suffering from diabetes or hypertension (compared to comorbid patients).

Clinical management index scores ranged from 5.15/10 ± 1.94 for hypertensive patients to 6.62/10 ± 1.7 for comorbid patients, with the average among all patients being 5.7/10. This flags a general lack of compliance with the international and national guidelines for diabetes and hypertension care and highlights a clear opportunity for improving the quality of care delivered to diabetic and hypertensive patients. This finding is particularly disconcerting in that it suggests a suboptimal control of diabetes and hypertension which increases substantively the risks of costly complications within the target population. This not only has consequences for patients and the course of their disease, but also threatens the capacities of a health system with already very scarce resources. The most critical areas of non-compliance indicated by the clinical management index were the referral to an ophthalmologist (only 16.9% of all patients referred), receiving most to all prescribed medications (only 15.7% patients reporting), and receiving smoking cessation advice (advised to only 44.2% of patients). It is also of concern that only three out of each five patients attended two GP visits per year as per the clinical management guidelines. These findings call for a deeper analysis of the root causes for non-compliance with the established guidelines and whether they relate to patients, care providers or the delivery system at large.

Our findings also flagged the sociodemographic characteristics of those in the target population receiving significantly poorer clinical management of their conditions. Those characteristics include being a Syrian refugee, a patient under 60 years of age, those who live in the urban setting of Beirut, those who have no formal schooling and those who benefit from free care.

Patients aged above 60 reported receiving better clinical management of their conditions compared to those belonging to younger age groups. One explanation for this could be that patients older than 60 become relieved from the financial burdens of healthcare access because they either move in with, or otherwise depend on, their children with higher current income or become eligible for health coverage (35). In Lebanon, a law was approved to extend the provision of health care to the entire population above the age of 64 through the National Social Security Fund (NSSF). This covers 90 per cent of hospitalization costs and 80 per cent of medical consultations and medication excluding dental care, while for Syrian refugees, the United Nations High Commissioner for Refugees (UNHCR) covers 85% of primary healthcare costs (36, 37).

Our results also illustrated the discrepancies on health outcomes by nationality. The health systems of countries receiving refugees are placed under tremendous pressure. Such systems often struggle to meet the urgent and acute health needs of the refugees, and as a result often neglect the care for patients with NCDs (38, 39). Refugees are specifically vulnerable to NCDs owing to several factors (38). The stress which results from fleeing one's home renders refugees susceptible to many chronic diseases such as hypertension, diabetes and many types of cancer (40, 41). In addition, refugees go through lifestyle changes which influence their dietary intake and activity levels and may as a result increase the risk of NCDs (38, 42). Earlier studies reported underutilization of NCDs services among Syrian refugees compared to Lebanese community members and showed that host community members had better access to care and fewer reports of medication interruption compared to refugees (43, 44). Study findings suggest that providing Syrian refugees with access to free or highly subsidized NCDs services is a necessary but not sufficient condition for them to be able to attain proper disease control. Many other determinants of health (e.g., income, education, employment, etc.) will negatively influence their health outcomes compared to their host communities.

Our results are also in accordance with other studies in demonstrating ties between socioeconomic status and health outcomes (35). People who belong to lower economic classes and who have less education are more likely to suffer from diseases, experience loss of functioning and experience higher mortality rates (35, 45). Education is a key determinant of health given that it influences both access to a range of resources such as income, safe neighborhoods, or healthier lifestyles (46). It also influences the attitudes and behaviors that lead to better health (35). People belonging to different socioeconomic groups lead different lifestyles in many aspects of life (e.g., childhood, educational experiences, work careers, marriage and family experiences, and health care) (47, 48).

Patients with health coverage received significantly better clinical management of their conditions compared to those with no health coverage. Given that health insurance is mostly provided by employers, people who lack health coverage are typically unemployed or have lower incomes (48, 49). Such socially disadvantaged patients have multiple risk factors (50). It is important to note that despite the presence of health coverage, patients would still be expected to make full or partial payment for some aspects of their care (e.g., drugs, lab tests). For example, patients covered by the NSSF still need to cover 20% of the cost of drugs and ambulatory care services. Our results demonstrated that patients that make partial or full payment for their medical expenses receive better quality of clinical care compared to patients who receive free healthcare. Patients benefiting from free care are usually the poorest and the most disadvantaged and, in the settings considered here, were primarily Syrian refugees and their families. In Lebanon, Syrian refugees benefit from free care through the 25 mobile medical units established by the Lebanese Ministry of Public Health in collaboration with UNHCR, NGOs and humanitarian agencies, which provide free consultations and medication to Syrian refugees. If access to a primary healthcare facility is unavailable, UNHCR covers 85% of primary healthcare (37). Disadvantaged Lebanese citizens with no health insurance resort to public hospitals or contracted private hospitals, where the Ministry of Public Health covers 95% and 85% of hospital care costs and 100% of medication costs for chronic and high-risk diseases (18, 51). While the provision of free healthcare is welcome and would improve accessibility to health service to NCDs patients, our findings suggest that a poorer quality of clinical management is reported by NCDs patients who are receiving free services. The results call on agencies providing free services to systematically monitor and evaluate the quality of such services since the subsidization of cost is a necessary but not sufficient condition for the equitable access to quality services by the vulnerable NCDs patients.

While each of the above-mentioned categories require targeted programming and attention, we argue that highest priority needs to be given to individuals with compounded vulnerability. For example, while being a Syrian refugee appears to negatively and significantly affect the quality of chronic care received, being an illiterate refugee seeking free care in Beirut will entail multiple layers of vulnerability and will require additional attention to ensure proper and equitable care for all patients. The findings thus call on donor agencies, NGOs and provider institutions to design targeted programs and activities that will ensure equitable delivery of services to diabetic and hypertensive patients with particular attention to patients with compounded vulnerability. While the context of Lebanon may be unique in some aspects, many of the recommendations in this paper would likely apply to other countries hosting a large number of refugees. The authors argue that the significant elements of vulnerability for NCD patients, including refugee status, literacy rate, and having health coverage, would apply to other contexts and recommend the carrying of studies similar to this one to validate the elements of vulnerability specific to each context.

The study has a number of shortcomings that are important to report. First, the QoC index, despite being grounded on the guidelines of multiple agencies (WHO, World Bank), best practice guidelines and the guidelines of the Ministry of Public Health, was never validated before and may need to be modified based on expert validation in the future. Despite strong assurances to the participating patients that their responses would not affect the care and/or aid they are receiving, it cannot be ascertained whether the study is free of bias toward poorer care and outcomes in anticipation of higher subsidies and continued support. The research team was not able to recruit equally from the two fragility contexts. The presence of a large number of refugees in fragility setting 2 have resulted in a larger number of responses from that setting. The fact that the study was planned and ethically approved prior to the COVID-19 pandemic, while data collection took place at the peak of the pandemic in Lebanon, did not allow the research team to systematically capture the effect of the pandemic on the quality of provided NCDs services to the target population. The pandemic also introduced a bias since people were reluctant to visit primary healthcare centers out of fear of contracting the virus. This may have caused a delay in seeking care and generally resulted in poor compliance to NCD protocols.

## Conclusion

The study identified a wide margin for improving quality of NCDs care in fragile contexts with particular gaps identified in referral to ophthalmology, accessing all prescribed medication and receiving counseling for smoking cessation. Additionally, findings indicate a number of predictors of comparatively poor quality of care that warrant attention, notably with regard to Syrian nationality/legal status, lack of health coverage, seeking free health provision and lower educational attachment. Although these are all relevant risk factors, the findings call on donor agencies, NGOs and provider institutions to design targeted programs and activities that especially ensure equitable delivery of services to diabetic and hypertensive patients with compounded vulnerability as a result of a number of these factors.

## Data availability statement

The datasets presented in this study can be found in online repositories. The names of the repository/repositories and accession number(s) can be found in the article/supplementary material.

## Ethics statement

The study protocol was reviewed and approved by the Ethics Research Panel of Queen Margaret University, Edinburgh (Protocol number QMU: REP 0201) and the Ethics Review Committee of the American University Beirut (protocol number AUB: SBS-2018-0514). The patients/participants provided their written informed consent to participate in this study.

## Author contributions

SS has made substantial contributions to the analysis, interpretation of the data, write up, and revising the manuscript. DM has made substantial contributions to the acquisition, analysis, interpretation of the data, drafting the original manuscript, and revising it. RH and AA have made substantial contributions to the interpretation of the data and the revision of the manuscript. HD has made substantial contributions to the analysis, interpretation of the data, and revising the manuscript. KD has made substantial contributions to the conception and design of the work, analysis, interpretation of the data, and revising the manuscript. SA has made substantial contributions to the analysis, interpretation of the data, and the revision of the manuscript. MA has made substantial contributions to the conception and design, supervision of the work, write up, and revision of the manuscript. All authors have read and approved the submitted version of the manuscript.

## Funding

This research was funded by the National Institute for Health Research (NIHR) Global Health Research program 16/136/100.

## Conflict of interest

The authors declare that the research was conducted in the absence of any commercial or financial relationships that could be construed as a potential conflict of interest.

## Publisher's note

All claims expressed in this article are solely those of the authors and do not necessarily represent those of their affiliated organizations, or those of the publisher, the editors and the reviewers. Any product that may be evaluated in this article, or claim that may be made by its manufacturer, is not guaranteed or endorsed by the publisher.

## Author disclaimer

The views expressed are those of the authors and not necessarily those of the National Health Service, the NIHR or the Department of Health and Social Care.
